# Dynamic failure characteristics of surrounding rocks under different lateral pressure coefficients in deep tunnel transient excavation

**DOI:** 10.1007/s40948-023-00563-x

**Published:** 2023-02-22

**Authors:** Ying Xu, Yuchao Yu, Wei Yao, Kaiwen Xia, Junxi Tang, Zhifeng Zhan

**Affiliations:** 1 State Key Laboratory of Hydraulic Engineering Simulation and Safety, School of Civil Engineering, Tianjin University, Tianjin, 300072 China; 2 State Key Laboratory of Explosion Science and Technology, Beijing Institute of Technology, Beijing, 100081 China; 3 Department of Civil and Mineral Engineering, University of Toronto, Toronto, M5S 1A4 Canada

**Keywords:** Deep tunnel, Transient excavation, Lateral pressure coefficient, PPV, Dynamic M–C criterion, EDZ

## Abstract

A novel transient unloading testing system was adopted to simulate the transient excavation of tunnels under different lateral pressure coefficients (*k*_0_). The results show that the transient excavation of a tunnel induces significant stress redistributions and concentrations, particle displacements and vibrations to the surrounding rocks. The decrease of *k*_0_ enhances the dynamic disturbance of transient tunnel excavation, and especially when *k*_0_ = 0.4 and 0.2, the tensile stress can be observed on the top of the tunnel. The peak particle velocity (PPV) of the measuring points on the top of the tunnel decreases with the increasing distance between the tunnel boundary and measuring point. The transient unloading wave is generally concentrated on lower frequencies in the amplitude-frequency spectrum under the same unloading conditions, especially for lower *k*_0_ values. In addition, the dynamic Mohr–Coulomb criterion was used to reveal the failure mechanism of a transient excavated tunnel by involving the loading rate effect. It is found that the excavation damaged zone (EDZ) of the tunnel is dominated by the shear failure, and the number of the shear failure zones increases with the decrease of *k*_0_. The EDZ of tunnels after transient excavations varies from ring-shape to egg-shape and X-type shear with the decrease of *k*_0_. The evolution of the EDZ induced by the transient unloading is associated with *k*_0_, i.e., the shear failure of surrounding rocks mainly occurs in the stress redistribution stage under high *k*_0_ (1.0–0.7), while the dramatic destruction of surrounding rocks is more prone to occur after the transient unloading process when *k*_0_ ≤ 0.6.

## Article Highlights

- Experimental methodology is developed to conduct transient excavation experiments.
- Transient excavation induces significant stress redistributions and concentrations, particle displacements and vibrations.
- Decrease in lateral pressure coefficient enhances the dynamic disturbance of transient tunnel excavation on surrounding rocks.
- The transient unloading wave can be obtained from the amplitude-frequency spectrum using FFT.
- Dynamic M–C criterion is developed to reveal the dynamic mechanism of a transient excavated tunnel.

## Introduction

The drill and blast tunneling (D&B) and tunnel boring machines (TBMs) (Ramoni and Anagnostou 2010) have been widely utilized for deep rock excavation. The safety of the excavation and construction in deep rock engineering is generally challenged by various geohazards, such as rockburst (Dowding and Andersson 1986; Keneti and Sainsbury 2018), rock slabbing and spalling (Kaiser and McCreath 1994; Gong et al 2012; Hidalgo and Nordlund 2012; Du et al 2016). In the past few years, considerable efforts have been made to reveal the mechanism of geohazards and design the corresponding preventive strategies (Dowding and Andersson 1986; Read et al 1998; Yang et al 2017a, b; Keneti and Sainsbury 2018; Xu et al 2022). However, the mechanism of these geohazards for deep tunnels is not yet mature due to the complicated geological conditions, such as the high in-situ stress, the high temperature (Zhao et al 2017), the complex lithological conditions such as the discontinuous faults and joints (Chakraborty et al 1994), and the brittle-ductile transition (Zhu et al 2022a, b) for deep rocks. Moreover, tunnel excavation leads to the transient release of in-situ stress and the violent vibration of the rock masses at the tunnel boundary (Lu et al 2012; Xie et al 2022), resulting in the generation of the excavation damage zone (EDZ) (Perras and Diederichs 2016a, b). Therefore, understanding the dynamic response and the failure mechanism of the surrounding rocks in a transient tunnel excavation is urgently needed.

Numerous efforts have been made to experimentally and theoretically address the failure mechanism and the evolution of the EDZ of the surrounding rocks in the past few decades (Martin 1993; Martini et al 1997; Sharan 2005; Gong et al 2012; Gao et al 2019; Zhu et al 2022a, b). The rapid stress redistribution (e.g., the tangential stress concentration) is one of the main reasons that contribute to the failure of rock masses (e.g., v-shaped notch) near the excavation boundary (Martini et al 1997). This failure can be approximately estimated by the theoretical solution of the stress concentration around a circular hole in a square plate (Timoshenko et al 1970) in combination with the failure criteria, i.e., the Mohr–Coulomb criterion (Mohr 1900), the Hoek–Brown criterion (Hoek and Brown 2019) and the Drucker–Prager criterion (Alejano and Bobet 2012). In addition, the brittle failure process was observed in in-situ tests in the deep tunneling (e.g., the Mine-by Experiment conducted by the AECL’s Underground Research Laboratory in Canada (Martini et al 1997), the KAERI underground research tunnel in Korea (Kwon et al 2009) and the Äspö Pillar Stability Experiment by the Äspö Hard Rock Laboratory in Sweden (Andersson et al 2009). Laboratory experiments (Haimson 2007; Labiouse et al 2014; Labiouse and Vietor 2014; Yang et al 2018; Li et al 2019) and theoretical and numerical simulations (Perras et al 2010; Perras and Diederichs 2016a, b; Vazaios et al 2019a, b; Sun et al 2021) were also conducted to explore the failure mechanism of the surrounding rocks. The lateral pressure coefficient can affect the shape and the dimension of the EDZ (Bai-quan et al 2015). Yi et al (2020) investigated the progressive fracture processes around tunnels triggered by blast disturbances under different lateral pressure coefficients and found that the influence of the disturbance is the smallest when the lateral pressure coefficient is 1.0. In addition, the shape of the tunnel (Zhong et al 2018; Gong et al 2022a, b), the excavation methods (Falls and Young 1998; Yang et al 2013) and the external disturbance (Jiang et al 2018; Jian-po et al 2020; Yi et al 2020) may also influence the formation of the EDZ.

However, the in-situ tests were commonly limited due to the high costs and the technique issues (Hartkorn 1997; Feng et al 2018). Also, the existing laboratory experimental methods cannot reproduce the tunnels’ real stress loading/unloading path (Sun et al 2021), especially for those excavated by D&B. The overloading tests were often conducted on the specimens with a prefabricated circular hole (Pan et al 2020; Yi et al 2020; Gong et al 2022a, b; Zhang et al 2022) or by excavating a hole in an intact rock mass under pre-stress (Yang et al 2018; Li et al 2019; Abierdi and Xiang 2020; Zhu et al 2020; Xiang et al 2021; Askaripour et al 2022; Gong et al 2022a, b; Hao et al 2022; Zhu et al 2022a, b). For model tests without an overloading procedure (Sun et al 2018; Xu et al 2021; Wang et al 2022), the EDZ is not as significant as expected, and thus the deformation and the stress redistribution of the surrounding rock masses are mainly discussed. In addition, the unloading rate of the laboratory experiments was lower than that in practice, e.g., excavating manually or by an electric drill (Haimson 2007; Yang et al 2018; Li et al 2019) or removing the oil pressure in a hollow cylinder specimen (Labiouse et al 2014; Labiouse and Vietor 2014). Thus, the failure mode obtained from these experiments cannot match well with the in-situ observation. Moreover, the theoretical and numerical analyses are efficient, but the dynamic process of the transient unloading in tunnel excavation is frequently neglected (Yang et al 2013; Zhao and Yang 2015; Ma et al 2020; Si and Gong 2020; Heidarzadeh et al 2021; Tu et al 2022), resulting in the underestimation of the compressive strength (Xia and Yao 2015; Wang et al 2020), the tensile strength (Wu et al 2015; Xia et al 2021) of the surrounding rocks and the overestimation of the EDZ. Therefore, the stress path, the unloading rate and the dynamic excavation process should be considered to experimentally reproduce the true tunnel excavation and obtain a reasonable theoretical and numerical solution, further revealing the dynamic response. Moreover, the failure mechanism of the surrounding rocks in a transient tunnel excavation should also be considered.

To achieve the research goals, a novel transient unloading testing was introduced and adopted in this study to simulate the transient excavation of tunnels under five different lateral pressure coefficients (*k*_0_ = 1.0, 0.8, 0.6, 0.4, 0.2). The stress redistributions and concentrations, particle displacements and vibrations on the surrounding rocks around the excavation were analyzed during the transient excavation. The peak particle velocity (PPV) around the boundary of the tunnel is discussed. The impact wave and the transient unloading wave were observed and analyzed using the amplitude-frequency spectrum through FFT. In addition, the dynamic Mohr–Coulomb criterion was used to reveal the failure mechanism of a transient excavated tunnel by involving the loading rate effect. Additionally, the geometry and the evolution of the EDZ of tunnels during transient excavations were determined under different lateral pressure coefficients.

## Experimental methodology and verification

### Experimental setup

**Fig. 1:**
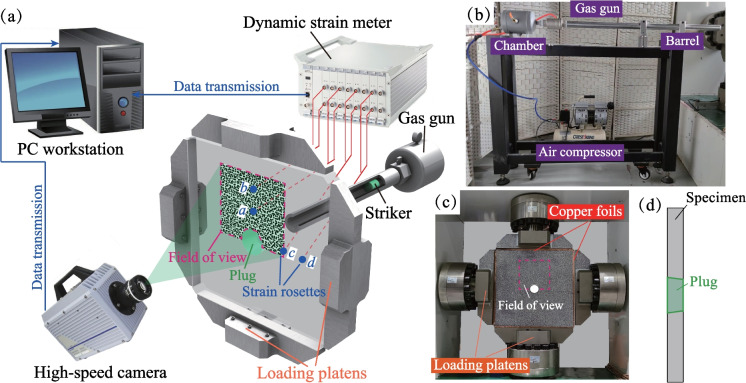
**a** Schematic of transient unloading testing system, **b** the impact unloading apparatus, **c** the biaxial loading frame and **d** the side view of the specimen and the plug (*Note a*_1_ and *a*_2_ denote the strain gauges parallel and perpendicular to the direction of major principal stress, which are used to measure the radial stress and tangential stress, respectively)

The transient unloading testing system for deep rocks is mainly composed of a biaxial loading frame, a data acquisition unit and an impact unloading apparatus (Fig. 1a and b). The details of the experiment system and the experimental procedures are thoroughly introduced by the authors (Yu et al 2022), and thus we will not add any detailed descriptions in this work. The impact unloading apparatus simulate the transient tunnel excavation process with a gas gun (Fig. 1b). To reduce friction in the interfaces, the edges of the PMMA specimen were wrapped with copper foils with a thickness of 0.3 mm (Fig. 1c), and the boundary of the prefabricated hole was greased with lubricant oil. The specimen is made of PMMA with a size of 520 mm × 520 mm × 20 mm (length × width × thickness), and the physical properties of PMMA are listed in Table 1. A hole with a 30 mm radius (*r* = 30 mm) was drilled in the center of the specimen and carefully processed into a truncated cone shape with a 5° base angle. A plug was specially designed to mimic the tunnel and polished into the truncated cone shape to ensure a snug fit with the prefabricated hole. The flexible design of the plug would weaken the interaction (e.g., the friction) at the specimen/plug interface and allows a high transient unloading speed of the tunnel (Yu et al 2022). Digital speckle patterns and four strain rosettes (named *a*, *b*, *c* and *d* in Fig. 1a) were arranged on the specimen for data acquisition.

**Table 1:** Physical parameters of PMMA

Density/*ρ*	Young’s modulus/*E*	Poisson’s ratio/*v*	P-wave velocity
1190 kg/m^3^	2.7 GPa	0.37	2.44 km/s

### The verification of the experimental method

Generally, the stress state of an unexcavated tunnel is under uniformed stresses, and the stress state of an excavated tunnel is equal to the theoretical solution of the stress/strain concentration around a circular hole (Timoshenko et al 1970):

1 $${\sigma }_{\rho }=\frac{{q}_{1}+{q}_{2}}{2}\left(1-\frac{{r}^{2}}{{\rho }^{2}}\right)+\frac{{q}_{1}-{q}_{2}}{2}\left(1-\frac{{r}^{2}}{{\rho }^{2}}\right)\left(1-3\frac{{r}^{2}}{{\rho }^{2}}\right)\mathrm{cos}2\varphi$$

2 $${\sigma }_{\varphi }=\frac{{q}_{1}+{q}_{2}}{2}\left(1+\frac{{r}^{2}}{{\rho }^{2}}\right)-\frac{{q}_{1}-{q}_{2}}{2}\left(1+3\frac{{r}^{4}}{{\rho }^{4}}\right)\mathrm{cos}2\varphi$$

where, *σ*_*ρ*_ and *σ*_*φ*_ are the radial and tangential stress of the surrounding rock masses (corresponding to the minimum principal stress *σ*_*3*_ and maximum principal stress *σ*_*1*_). *q*_1_, *q*_2_ are the vertical and horizontal load of the platen, *r* is the radius of the prefabricated hole, *ρ* and *φ* are the polar radius and angle of the point. Therefore, the change magnitude of the radial and tangential stress (named as the normalized radial stress (*σ*_*ρ*_/*q*_1_, NRS) and normalized tangential stress (*σ*_*φ*_/*q*_2_, NTS), respectively) can be used to evaluate the efficiency of the proposed experimental method if the dynamic stress process is neglected. For example, for locations at (2*r*, 0°) and (3*r*, 90°) and lateral pressure coefficient of 1.0 (*q*_2_/*q*_1_ = 1.0), the NRS and NTS are 0.75, 1.25 and 0.89, 1.11, respectively. In this work, the transient unloading experiments with *k*_0_ = 1.0 were conducted by adjusting the horizontal and vertical loads to 80 kN.

**Fig. 2:**
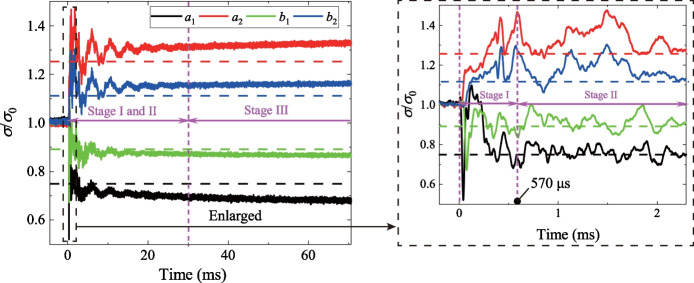
Comparison of normalized stresses with the theoretical solutions

**Fig. 3:**
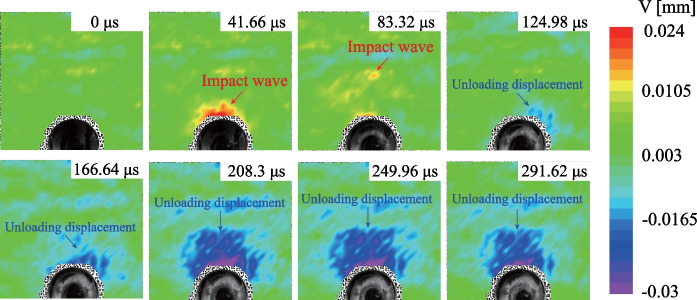
The vertical unloading displacement of the surrounding rocks

The comparison between the normalized stresses (solid lines) and the theoretical solutions (dashed lines) is shown in Fig. 2. Notably, the experimental results are consistent with the theoretical solutions. The process of stress redistribution at the measuring points could be roughly divided into three stages: (I) rapid stress redistribution, (II) stress transition stage with fluctuation and (III) stable stress state (Yu et al 2022). In Stage I, the radial and tangential stresses dramatically changed as the transient unloading was completed. The vertical displacement of the surrounding rocks derived from the DIC analysis (Fig. 3) also demonstrates that the transient excavation through the plug causes a considerable unloading process for the surrounding rocks. A compressive impact wave was first detected (41.66 μs in Fig. 3). Then the unloading displacements of the surrounding rocks were dramatically changed (124.98 μs in Fig. 3). In addition, the stresses on the strain rosettes experienced oscillations in Stage II, and the oscillations at the tangential stresses were more extensive than those of the radial stresses (Fig. 2). The oscillations of the specimen were gradually diminished and then followed by Stage III. The internal stresses of the specimen remained stable in the third stage. Accordingly, when *k*_0_ = 1.0, the duration of Stage I is 570 μs (Fig. 2) and the stress change of gage *a*_1_ is 0.674 MPa. The strain rate of the transient unloading can be estimated by 0.674 MPa/570 μs/2.7 GPa = 0.4377 s^−1^, indicating that the transient unloading of a tunnel specimen in our study is a dynamic process (Lu et al 2012; Yang et al 2017a, b). Therefore, the proposed experimental method can efficiently reproduce the transient excavation of a deep-buried tunnel and the dynamic processes of the stress redistribution of the surrounding rocks during the transient unloading.

## The dynamic mechanism of a transient excavated tunnel under different lateral pressure coefficients

The transient unloading process of tunnel excavation under five lateral pressure coefficient levels (*k*_0_ = 1.0, 0.8, 0.6, 0.4, 0.2) was observed. It is noted that the forces of the vertical platens *F*_v_ were fixed at 80 kN, and the forces of the horizontal platens *F*_h_ were changed accordingly to achieve the predetermined lateral pressure coefficient *k*_0_. For example, a lateral pressure coefficient of 0.4 means *F*_v_ = 80 kN and *F*_h_ = 32 kN. Another notable feature here is that the air pressure of the impact unloading apparatus is fixed at 10 psi (~ 0.069 MPa), meaning that the velocity of the striker is constant for all experiments.

### The stress redistribution of the surrounding rocks

**Fig. 4:**
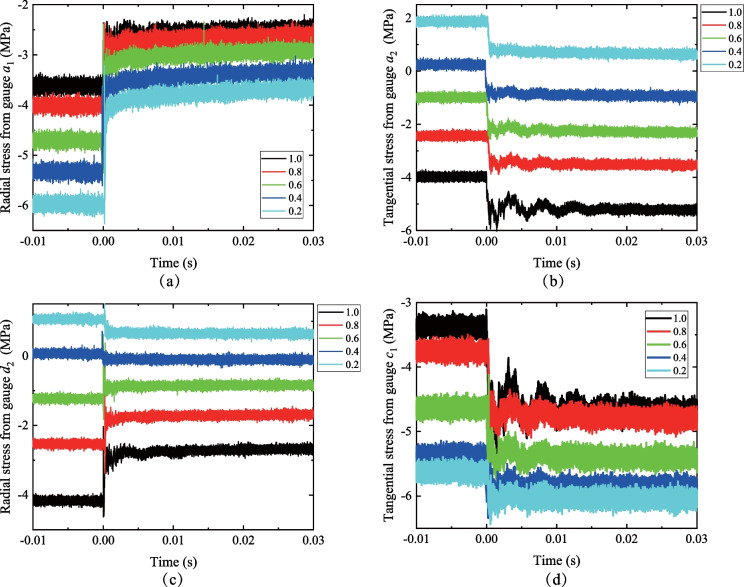
The stress redistribution recorded from the strain rosettes **a** *a*_1_, **b** *a*_2_, **c** *c*_1_ and **d** *c*_2_ (The negative values are given for the compressive stress)

The stress redistribution (i.e., the stress concentration or the stress relaxation) of the surrounding rocks is crucial to reveal the failure mechanism of an excavated deep tunnel. It is well known that the tangential and radial stresses are the maximum principal stress *σ*_1_ and the minimum principal stress *σ*_3_ of an in-plane measuring point, respectively. The stress redistribution recorded by the strain rosettes *a* and *c* under different lateral pressure coefficients are shown in Fig. 4. The stress redistribution of the gauges on the top (*a*_1_, *a*_2_) and the vault (*c*_1_, *c*_2_) of the tunnel is dramatically changed with the lateral pressure coefficients. The initial value of the radial stress from gauge *a*_1_ (Fig. 4a) is negative and decreases with a decreasing *k*_0_ value (i.e., from 1.0 to 0.6), while the decrement of the stresses before and after the transient unloading gradually decreases. The initial value of the tangential stress from gauge *a*_2_ (Fig. 4b) decreases from positive to negative with the increase of *k*_0_, and the tangential stress at gauge *a*_2_ changes from the tension to the compression when *k*_0_ exceeds 0.4. For the strain rosette *c*_1_ (Fig. 4c), the initial value of the radial stress turns from the tension to the compression when *k*_0_ is larger than 0.4. Moreover, the variation of the radial stress increases from a negative to positive value, indicating that the radial stress decreases with the increase of *k*_0_ regardless of the sign of the stress when *k*_0_ = 1.0. The tangential stress from gauge *c*_2_ (Fig. 4d) is negative and increases with the increase of *k*_0_. The variation of the tangential stress of *c*_2_ also increases with the increase of *k*_0_. Therefore, the tensile stress at the surrounding rocks near an excavated tunnel boundary is generated in the direction of the minimum principal stress when *k*_0_ is smaller than 0.4.

**Fig. 5:**
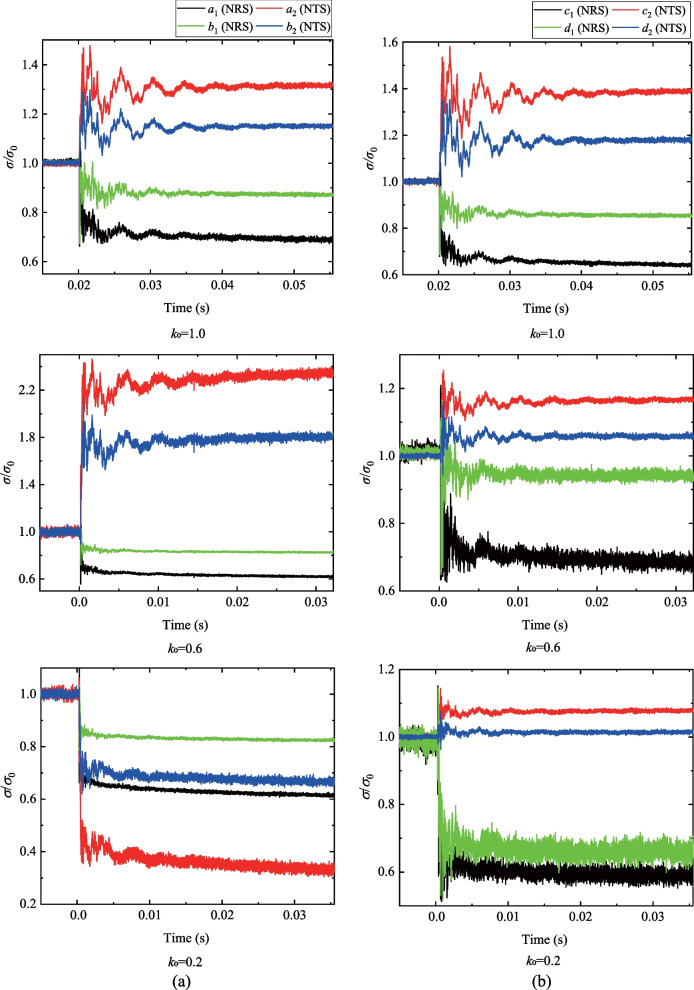
The stress redistribution recorded by **a** strain rosettes *a* and *b*, and by **b** strain rosettes *c* and *d* (NRS: normalized radial stress; NTS: normalized tangential stress)

**Fig. 6:**
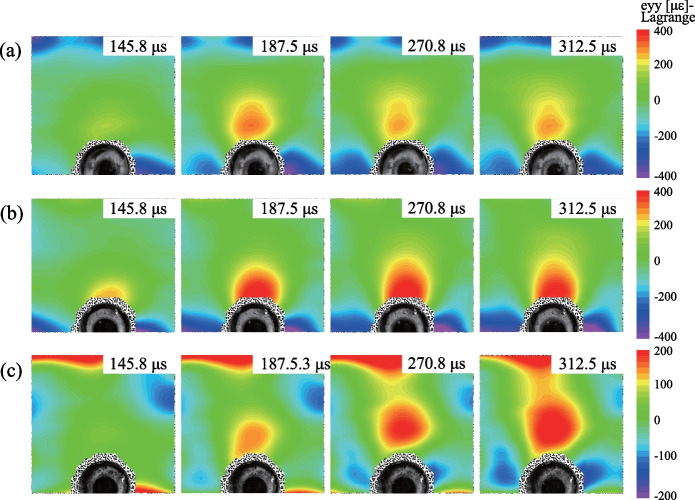
The evolution of vertical micro-strain of the surrounding rock of a transient excavated tunnel under **a** *k*_0_ = 1.0; **b** *k*_0_ = 0.6; **c** *k*_0_ = 0.2 (The negative value denotes the compressive strain)

The stresses obtained from the strain rosettes (i.e., *a*, *b*, *c* and *d*) were normalized in Fig. 5. The normalized radial stress (NRS) of gauges *a*_1_ and *b*_1_ decreases after the transient excavation/unloading and the magnitude of the reduction increases with the decrease of lateral pressure coefficient *k*_0_, i.e., NRS decreases from 0.69 to 0.61 for strain gauge *a*_1_ and decreases from 0.87 to 0.85 for stain gauge *b*_1_. However, the normalized tangential stresses (NTS) variation for strain gauges *a*_2_ and *b*_2_ increases as the lateral pressure coefficient decreases from 1.0 to 0.6, i.e., NTS increases from 1.3 to 2.2 for strain gauge *a*_2_ and increases from 1.15 to 1.8 for strain gauge *b*_2_. When *k*_0_ decreases to 0.2, both the NRS and NTS decrease after the transient excavation/unloading (Fig. 5a). For strain rosettes *c* and *d* (Fig. 5b), the NTS (i.e., *c*_2_ and *d*_2_) are generally found to increase with the lateral pressure coefficient *k*_0_ and the variation increases with the increase of *k*_0_. The NRS of the strain gauges *c*_1_ and *d*_1_ decreases as *k*_0_ decreases from 1.0 to 0.6. The evolution of the vertical micro-strain of the surrounding rock of a transient excavated tunnel under different lateral pressure coefficients (*k*_0_ = 1.0, 0.6, 0.2) is plotted in Fig. 6. The stress concentration in the vertical direction was detected in the vaults of the excavated tunnels. The domain of the stress concentration changes with the decrease of *k*_0_, i.e., the domain of the compressive stress concentration is flatter at *k*_0_ = 0.6 (Fig. 6b) than that at *k*_0_ = 1.0 (Fig. 6a) and changes into a long and inclined geometry when *k*_0_ = 0.2 (Fig. 6c). It can be concluded that the transient unloading process (e.g., the stress redistribution) of the surrounding rocks is affected by the stress state applied to the specimen (i.e., lateral pressure coefficient *k*_0_). The radial stress of the surrounding rocks decreases after transient excavation regardless of the value of lateral pressure coefficient *k*_0_ (Figs. 4 and 6), while the variation of the tangential stress depends on both the value of *k*_0_ and the location of the surrounding rocks (i.e., the top/bottom or the vault of the tunnel). For the measuring points on the top of the tunnel (e.g., strain rosettes *a* and *b*), the tangential stress increases with the increase of *k*_0_; for the measuring points on the tunnel vaults (e.g., strain rosettes *c* and *d*), the tangential stress increases when *k*_0_ is greater than 0.4 and decreases when *k*_0_ is less than 0.4 (Fig. 4). Moreover, the tensile stress can be observed at the top of the tunnel (parallel to the direction of minor principal stress) when the lateral pressure coefficient *k*_0_ is smaller than 0.4. Therefore, the risks of tensile failure for an excavated tunnel under the in-situ stress increase as the decrease of lateral pressure coefficient *k*_0_. Furthermore, tensile failures are commonly observed with shear failures in a bias tunnel (Martini et al 1997; Vazaios et al 2019a, b).

### The evolution of particle displacement

**Fig. 7:**
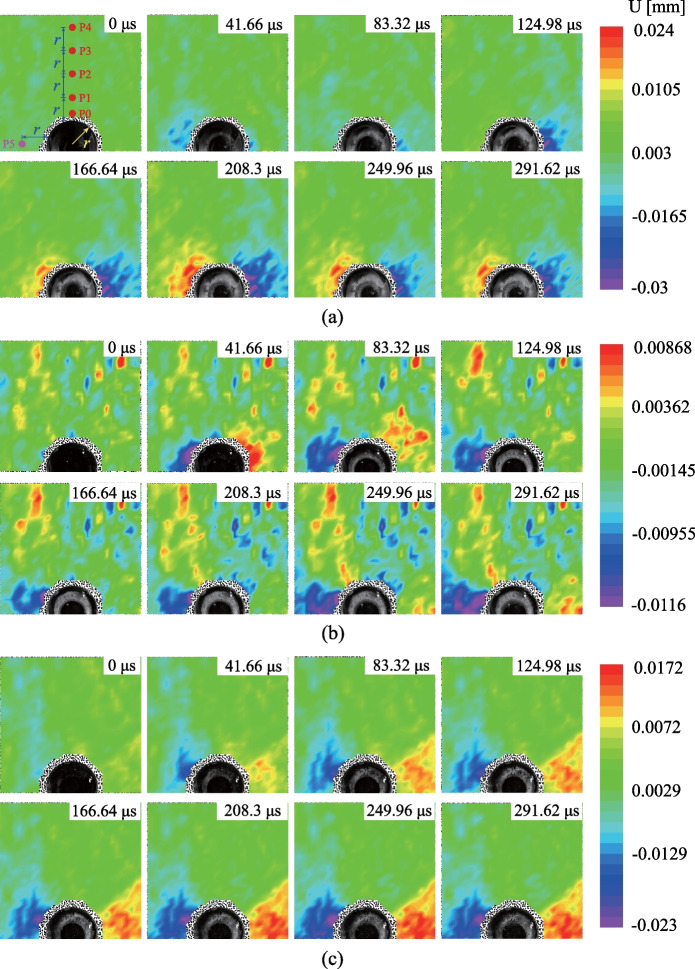
The evolution of horizontal displacement (U) of the surrounding rocks: **a** *k*_0_ = 1.0, **b** *k*_0_ = 0.6, **c** *k*_0_ = 0.2

**Fig. 8:**
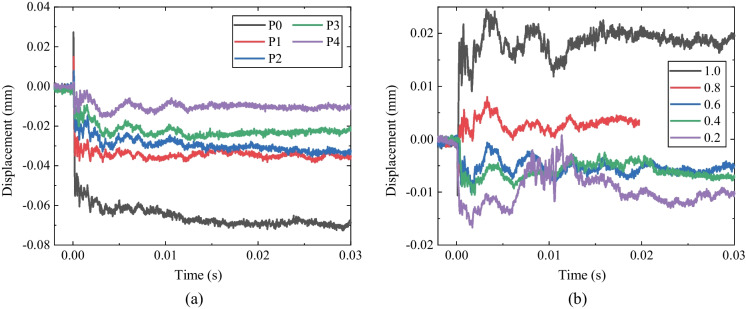
**a** The vertical displacement of the measuring points P0-P4 when *k*_0_ = 1.0; **b** The horizontal displacement of P5 under different *k*_0_

The evolution of the particle displacement is a crucial factor in evaluating the dynamic mechanism of the transient unloading in deep rock excavation. According to our previous discussion, it can be inferred that the vertical displacement distribution of the surrounding rocks is generally similar to that in Fig. 3, in which the particle movement on the top of the tunnel is negative (i.e., downwards to the center of the tunnel). Moreover, it is found that the variation of the tangential displacement of the surrounding rocks is much smaller than the radial displacement and there seems to be no significant relationship between the tangential displacement and the lateral pressure coefficient. This demonstrates that the influence of the transient unloading on the surrounding rocks mainly focuses on radial deformation. Figure 7 shows the evolution of the horizontal displacement (U) of the surrounding rocks with the *k*_0_ = 1.0, 0.6 and 0.2, respectively. One can see that the maximum horizontal displacement occurs in the direction of the minimum principal stress near the tunnel edge. The vertical displacements of the measuring points P0–P4 when *k*_0_ = 1.0 are shown in Fig. 8a. The vertical displacement is negative and decreases with the distance between the measuring point and the center of the tunnel. This is consistent with the experimental observations on the tunnel subjected to step-by-step excavation in previous studies (Yang et al 2018; Zhu et al 2020; Zhu et al 2022a, b). Moreover, the horizontal displacements of the particles on the vaults of the tunnel are positive (i.e., towards the tunnel center) when *k*_0_ = 1.0 (Fig. 7a), and its sign changes when *k*_0_ is smaller than 0.6 (Fig. 7b and c). Further, the horizontal displacement of P5 was derived from the DIC analysis and is shown in Fig. 8b. The horizontal displacement of P5 is positive (rightwards). It turns negative when *k*_0_ is smaller than 0.6, indicating that the transient excavation of the tunnel leads to the tensile stress on the surrounding rocks when the lateral pressure coefficient is smaller than 0.6, which is consistent with the previous analysis of the stress redistribution of the surrounding rocks.

### The evolution of particle vibration velocity

**Fig. 9:**
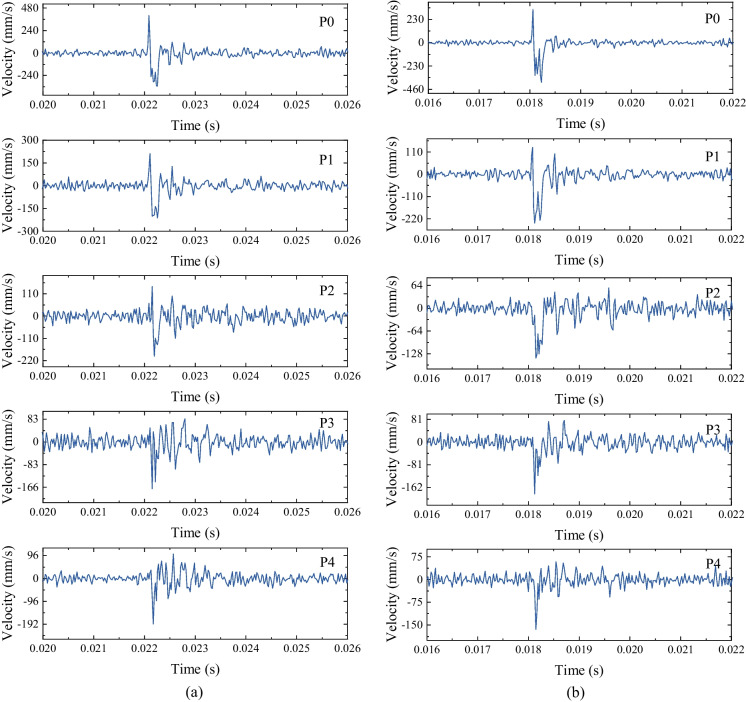
The velocity spectrum of the vertical measuring points: **a** *k*_0_ = 1.0; **b** *k*_0_ = 0.4

**Fig. 10:**
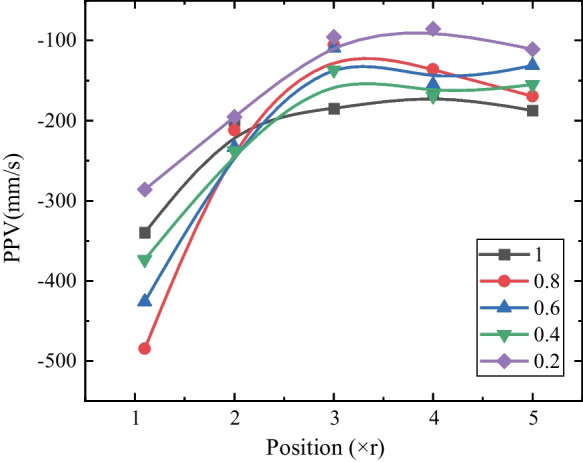
Vertical PPVs of the measuring points under different lateral pressure coefficients

The vibration of the surrounding rocks induced by the transient tunnel excavation is valuable in guiding construction and blasting design in engineering practice (Yang et al 2016, 2019). Thus, to further investigate the dynamic mechanism of transient unloading, we extracted the displacement history curves of the points in the vertical measuring line (i.e., P0–P4) and obtained the radial vibration velocity spectrum of the corresponding points (Fig. 9). The compressive impact wave (i.e., the positive part) was detected in the front of the unloading wave (i.e., the negative part), and the amplitude of the impact wave tends to decrease during the propagation. For instance, the positive parts almost disappear at P3 (i.e., 3*r* from the excavating tunnel boundary). Moreover, the amplitude of the unloading pulse (i.e., the negative part) decreases with the increase of the propagation distance, e.g., the peak particle velocity (PPV) decreases from 339.98 (P0) to 166.25 mm/s (P3) when *k*_0_ = 1.0 (Fig. 9a) and from 373.33 (P0) to 169.09 mm/s (P3) when *k*_0_ = 0.4 (Fig. 9b). The PPVs of P0-P4 under different lateral pressure coefficients are plotted in Fig. 10. The PPVs of the measuring points (i.e., P0, P1 and P2) decrease as the points move away from the hole and it is also observed that the PPVs are identical when the distance exceeds 4*r* (i.e., P3 and P4).

**Fig. 11:**
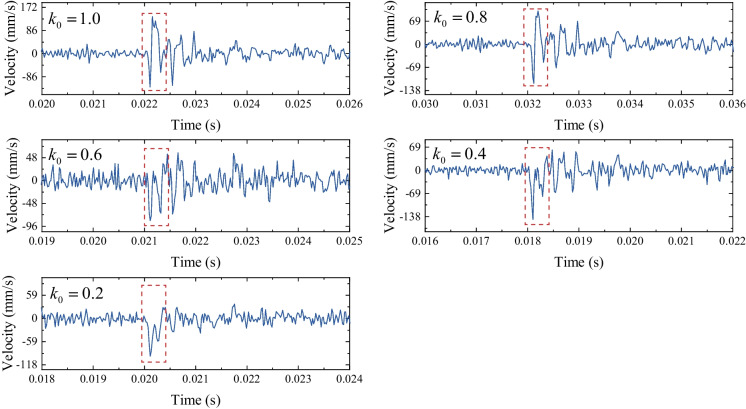
The velocity spectrum of the horizontal measuring point P5 under different *k*_0_

**Fig. 12:**
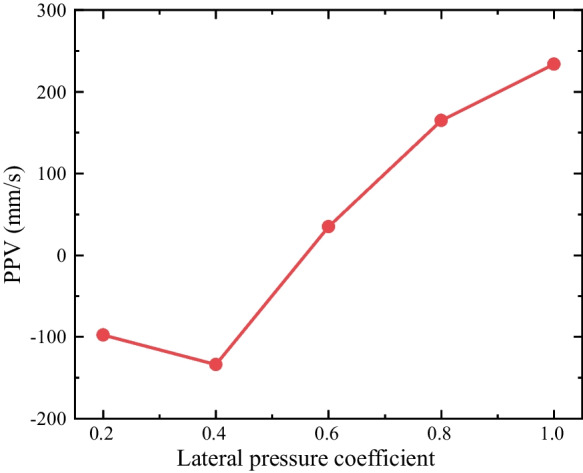
Horizontal PPV of P5 under different lateral pressure coefficients

The velocity spectrums of the horizontal measuring point P5 under the different lateral pressure coefficients (i.e., 1.0, 0.8, 0.6, 0.4 and 0.2) are plotted in Fig. 11. The compressive impact waves are also observed in the velocity spectrums, and the PPVs changes from a positive value to a negative value (i.e., a negative speed indicates a leftward movement of a horizontal measuring point) as the *k*_0_ decrease from 1.0 to 0.2. The PPVs of P5 under different lateral pressure coefficients were derived from Fig. 11 and plotted in Fig. 12. It demonstrates that the particles on the vault of the tunnel (i.e., the location of P5) start to move away from the hole when *k*_0_ is smaller than 0.6.

### The analysis of the amplitude spectrum

**Fig. 13:**
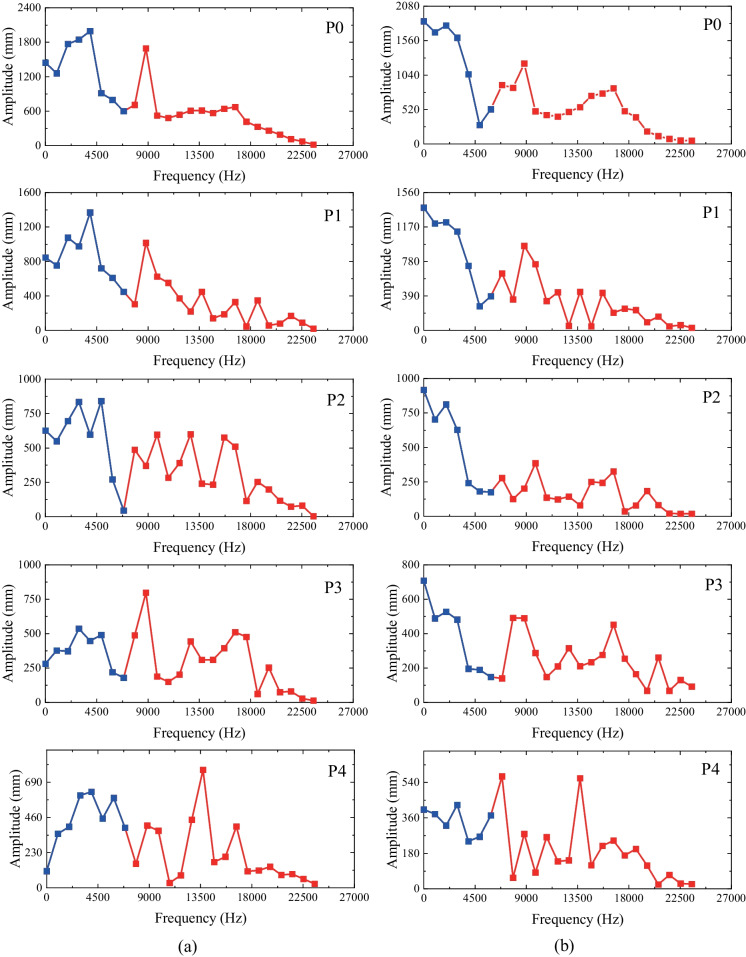
The amplitude-frequency spectrum of the surrounding rocks after FFT: **a** *k*_0_ = 1.0; **b** *k*_0_ = 0.4

In the micro-seismic monitoring of tunnel excavation in rock engineering practices, the micro-seismic waves are generally regarded as the superposition of the blasting load and the transient release of in-situ stress. The relatively low-frequency components were believed to be associated with the transient release of in-situ stress (Yang et al 2013, 2019). As discussed above, the transient unloading method in this paper can reproduce the transient unloading stress path and record the surrounding rocks’ dynamic response (i.e., the displacement and the particle vibration). Based on the detected stress redistribution data (Figs. 4, 5, 6), the displacement contour (Figs. 7 and 8) and the velocity spectrum of the measuring points (Figs. 9, 10, 11), the transient unloading signals tightly follows the impact wave generated by the impact between the striker and the plug. Due to the difficulty in identifying these two types of vibrations, the amplitude-frequency spectrum of the velocity spectrum was obtained through the Fast Fourier Transform (FFT). As shown in Fig. 13, the primary frequency of the transient unloading wave and the impact wave are 3800 Hz and 8769 Hz, and the inflection frequency is in the range of 6780–7850 Hz (Fig. 13a). Moreover, the amplitude of the amplitude-frequency spectrum decreases as the distance between the measuring point and the tunnel center increases. Besides, the low-frequency components in the frequency-amplitude spectrum increase significantly as the lateral pressure coefficient decreases. For example, the greater amplitudes are mainly distributed in 0–6780 Hz when the lateral pressure coefficient is 1.0. Regarding a lower lateral pressure coefficient (*k*_0_ = 0.4), the greater frequency is concentrated in 0–4500 Hz (Fig. 13b). Therefore, under the same excavation condition, the reduction of the lateral pressure coefficient can increase the amplitude in a lower frequency range of the amplitude-frequency spectrum. Due to the low natural frequency of underground structures, if the frequency band of the larger amplitude component in the unloading wave is low, it is more likely to cause structure resonance (Wu et al 2005; Cheng et al 2022).

## The dynamic failure mechanism of a transient excavated tunnel

### The development of the dynamic Mohr–Coulomb failure criterion

**Fig. 14:**
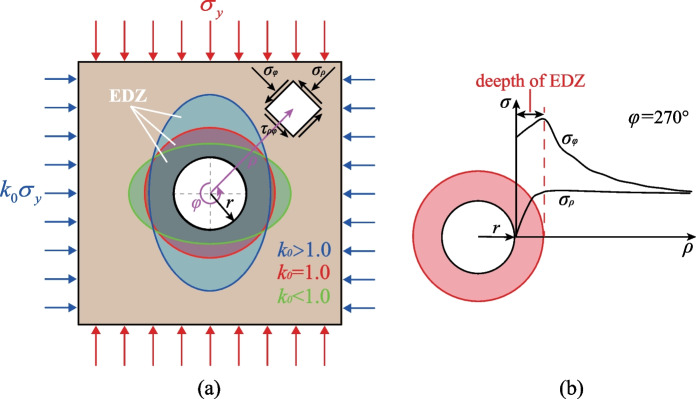
Schematic of an excavated tunnel: **a** the distribution of EDZ; **b** The stress state of the surrounding rocks

The transient tunnel excavation significantly results in stress redistribution, particle displacement and vibration of the surrounding rocks. If the in-situ stress is large enough, the destructive failure can be induced in the surrounding rocks following the transient tunnel excavation, further causing the expansion of the excavation damage zone (EDZ) (Perras et al 2010; Perras and Diederichs 2016a, b; Vazaios et al 2019a, b), leading to geohazards such as rock slabbing and rockburst. The envelope of EDZ and the stress state of the surrounding rocks of an excavated tunnel are shown in Fig. 14a. The geometry of EDZ is influenced by the lateral pressure coefficients *k*_0_, while the EDZ affects the tangential and the radial stress of the surrounding rocks (Fig. 14b).

**Fig. 15:**
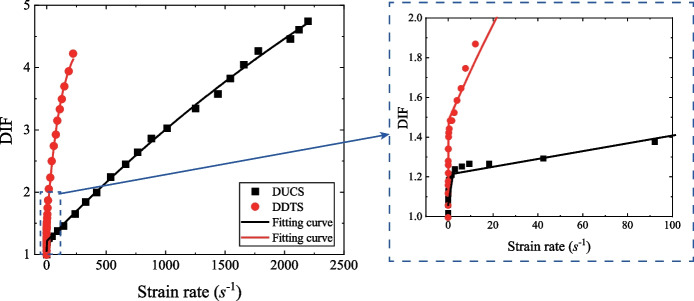
The strain rate dependency of the dynamic compressive and direct tensile strengths [after Zhang and Zhao (2014)]

To better understand the failure mechanism of the surrounding rocks of the tunnel, the Mohr–Coulomb (M–C) criterion (Coulomb 1773; Mohr 1900) was introduced to address the failure mechanism of the surrounding rocks and the evolution of EDZ of the tunnel since it has been extensively used in the theoretical and numerical analysis (Yang and Yin 2004; Labuz and Zang 2012; Yao et al 2017). The shear failure and the tensile failure of the material can be determined by the M–C failure criterion and the maximum tensile stress criterion through *σ*_1_ and *σ*_3_:

3 $${f}^{s}={\sigma }_{1}-\frac{1+sin\varphi }{1-sin\varphi }{\sigma }_{3}-\frac{2c\mathrm{cos}\varphi }{1-sin\varphi }\ge 0$$

4 $${f}^{t}={\sigma }_{3}+{\sigma }_{t}\le 0$$

where, *σ*_*t*_ is the tensile strength, *c* and *φ* are the cohesion and the internal friction angle of the material. Equations (3) and (4) are generally suitable to estimate the failure of the rocks under static loading conditions, whereas rocks in the transient unloading processes are subjected to dynamic loads. Meanwhile, the tensile and shear strength of rocks are significantly rate-dependent, and therefore, it is necessary to modify Eqs. (3) and (4) by considering the rate-dependency on the tensile strength *σ*_*t*_ and the dynamic shear properties (i.e., *c* and *φ*) of rocks. The dynamic increase factor (DIF) is defined as the ratio of the dynamic compression/tension strength to the corresponding quasi-static compression/tension strength for rocks (Liu et al 2018). It has been widely used to evaluate the dynamic mechanical properties by using the static mechanical properties of rocks. Based on the experimental data in the existing study (Zhang and Zhao 2014), the DIF for the dynamic uniaxial compressive strength (DUCS) and the dynamic direct tensile strength (DDTS) for rocks in terms of the strain rate can be fitted by the following exponential function (as shown in Fig. 15):

5 $$DIF=a+b\left(1-{e}^{-\frac{\dot{\varepsilon }\left(t\right)}{{\dot{\varepsilon }}_{1}}}\right)+c\left(1-{e}^{-\frac{\dot{\varepsilon }\left(t\right)}{{\dot{\varepsilon }}_{2}}}\right)$$

where, *a*, *b*, *c*, ${\dot{\varepsilon }}_{1}$ and ${\dot{\varepsilon }}_{2}$ are the fitting parameters. It should be noted that DIF = *a* ≈ 1.0 when $\dot{\upvarepsilon }\left(t\right)=0$ and a limit of the DIF (= *a* + *b* + *c*) can be reached when $\dot{\varepsilon }\left(t\right)$ is large enough. These parameters for both DUCS and DDTS are listed in Table 2. The limit of the DIF for the dynamic tensile strength and the compressive strength can be estimated as 4.607 and 10.754, indicating the dynamic strengths cannot infinitely increase with the strain rates, and this function is reasonable.

**Table 2:** The fitting parameters of DIF

DIF	*a*	*b*	*c*	${\dot{\upvarepsilon }}_{1}$	${\dot{\upvarepsilon }}_{2}$	R-squared
DUCS	1.058	0.153	9.543	0.055	4798.713	0.999
DDTS	1.110	0.371	3.126	0.064	118.241	0.997

Based on Eq. (5), the modification of the dynamic tensile strength in the dynamic M–C failure criterion can be obtained:

6 $${f}^{t}\left(t\right)={\sigma }_{3}+{\sigma }_{t}\left(t\right)={\sigma }_{3}+{\sigma }_{t0}\left[a+b\left(1-{e}^{-\frac{\dot{\varepsilon }\left(t\right)}{{\dot{\varepsilon }}_{1}}}\right)+c\left(1-{e}^{-\frac{\dot{\varepsilon }\left(t\right)}{{\dot{\varepsilon }}_{2}}}\right)\right]\le 0$$

In addition, the dynamic shear strength in the M–C failure criterion is mainly determined by the cohesion *c* and internal friction angle *φ* (Eq. (3)). The internal friction angle *φ* can be considered to be constant for rocks under dynamic loading conditions (Huang et al 2012; Yao et al 2017). Therefore, the determination for the dynamic shear failure can be rewritten by substituting Eq. (5) into Eq. (3):

7 $$\begin{aligned} f^{s} \left( t \right) & = \sigma_{1} - \frac{1 + sin\varphi }{{1 - sin\varphi }}\sigma_{3} - \frac{1 + sin\varphi }{{1 - sin\varphi }}\sigma_{t} \left( t \right) \\ & = \sigma_{1} - \frac{1 + sin\varphi }{{1 - sin\varphi }}\sigma_{3} - \frac{1 + sin\varphi }{{1 - sin\varphi }}\sigma_{t0} \left[ {a + b\left( {1 - e^{{ - \frac{{\dot{\varepsilon }\left( t \right)}}{{\dot{\varepsilon }_{1} }}}} } \right) + c\left( {1 - e^{{ - \frac{{\dot{\varepsilon }\left( t \right)}}{{\dot{\varepsilon }_{2} }}}} } \right)} \right] \le 0 \\ \end{aligned}$$

### The realization of transient excavation of a tunnel using the FLAC3D program

**Fig. 16:**
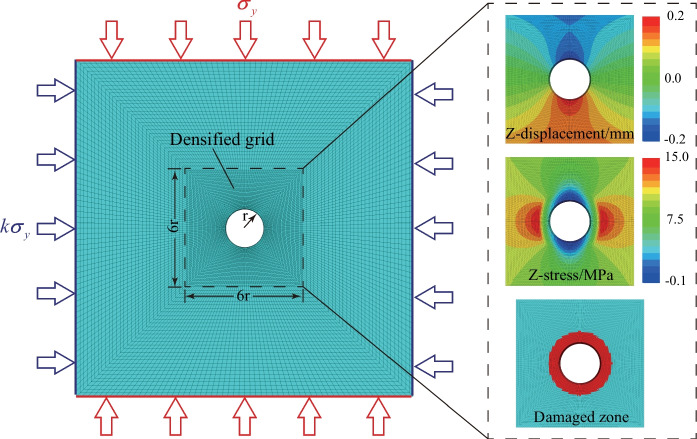
The geometry and the boundary conditions of the 2D numerical model and the basic simulation results of the surrounding rocks

A numerical model with the same size as the PMMA specimen (520 mm × 520 mm × 20 mm) and a 60 mm diameter prefabricated hole was established using the FLAC3D program, as shown in Fig. 16 (Sitharam et al 2007; Wang et al 2012; Bai et al 2022). The numerical model consists of 14,400 zones, and the zones near the hole with a size of 180 mm × 180 mm were densified for better observation. The isotropic elastic model was assigned to obtain the deformation and stress fields of the numerical model, and the dynamic Mohr–Coulomb failure criterion mentioned above [Eq. (7)] was introduced into the model to evaluate the failure characteristic and process of the transient excavated tunnel. The elastic parameters in this numerical model were consistent with those of the PMMA (Table 1) to better compare the numerical results with the experimental results. However, to effectively analyze the failure and damage of the surrounding rocks during the transient unloading process, the parameters (i.e., *σ*_*t*_, *c* and *φ*) for determining the material’s tensile and shear failure behaviors are given in Table 3.

**Table 3:** The physical and mechanical parameters of the model

Density *ρ*	Young’s modulus *E*	Poisson’s ratio *v*	Cohesion *c*	Friction *φ*	Tensile strength *σ*_*t*_	Compressive strength *σ*_*c*_
1190 kg/m^3^	2.7 GPa	0.37	1 MPa	35°	1.04 MPa	6.24 MPa

**Fig. 17:**
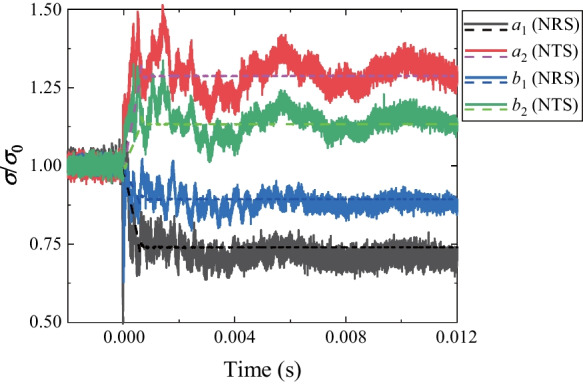
Comparison of the stress history curves between the experimental (solid lines) and numerical (dashed lines) results (*k*_0_ = 1.0)

To mimic the unloading process, an excavation relaxation method proposed by Cai et al. (2008) was introduced by reducing Young’s modulus of the excavating area. It is worth noting that excavating by directly deleting the zones within the excavating area is similar to the excavation relaxation method if the target Young’s modulus is set to 0 GPa. Therefore, the young’s modulus in the damaged zone was set to 1/20 of the initial value (Table 3) in this work. Additionally, previous studies have suggested removing the supporting forces at the excavation boundary to simulate the unloading process. Therefore, in this study, the transient unloading process was achieved by Fish programming in the following steps: (a) the desired biaxial loads were exerted on the model without the hole (i.e., tunnel) and the group zones of the tunnel were then removed. The numerical model was run for one step to obtain the real-time forces of the grid points on the excavation boundary; (b) these forces were stored in an array, and then the equal and opposite forces were applied to the grid points at the excavation boundary. After that, the displacement and the velocity of the model were cleared to maintain the efficiency of the continuing calculation; (c) The external forces within a particular duration (the exact duration in Stage I obtained from the experiments in Fig. 9) were removed accordingly. Finally, the model was run until the expansion of the EDZ was stopped. Notably, the duration of stress redistribution in Stage I was considered in the numerical simulation. The normalized stress history curves [i.e., NRS (*σ*_*ρ*_/*σ*_*0*_) and NTS (*σ*_*φ*_/*σ*_*0*_)] between the numerical and experimental results are compared in Fig. 17. The excellent consistency between the experimental and the numerical simulation results can be observed. As shown in Fig. 17, the NRS and NTS histories in the numerical simulation are almost identical to the experimental curves. Therefore, this numerical model is valid for reproducing the unloading stress path of a transient excavated tunnel.

### The failure mechanism of a transient excavated tunnel under various lateral coefficients

**Fig. 18:**
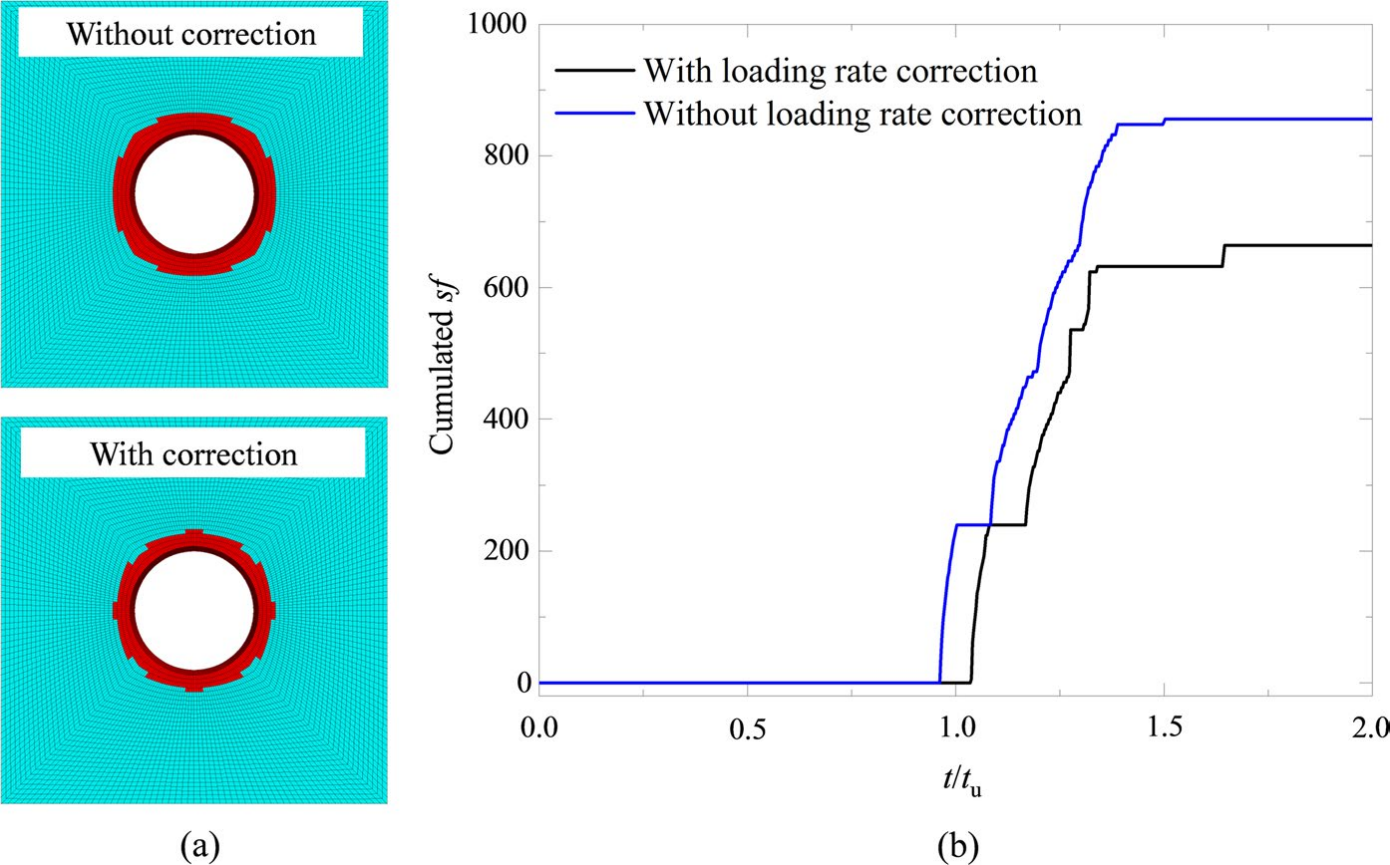
**a** The distribution of EDZ of a transient excavated tunnel without and with the loading rate correction and **b** The cumulated *sf* of the transient excavated tunnel

The dynamic failure mechanism of a transient excavated tunnel under various lateral coefficients (*k*_0_ = 1.0, 0.9, 0.8, 0.7, 0.6, 0.5, 0.4, 0.3) was explored using the numerical model in Fig. 16. To illustrate the function of the loading rate correction [Eq. (7)], we compared the distribution of EDZ of a transient excavated tunnel without the loading rate correction and with the loading rate correction in Fig. 18a when *t*/*t*_u_ = 2.0. As shown, the EDZ without the loading rate correction is larger than that with the loading rate correction, indicating that the dynamic correction of the model parameters can enhance the capacity of the surrounding rocks. Moreover, we counted the cumulated shear failure zone number *sf* with and without the loading rate correction (Fig. 18b). One can see that the *sf* number without the loading rate correction is larger than that with the loading rate correction, and the failure onset of the model without the loading rate correction is prior to that with the loading rate correction. Therefore, the loading rate correction is important in providing accurate responses of surrounding rocks of a transient excavated tunnel.

**Fig. 19:**
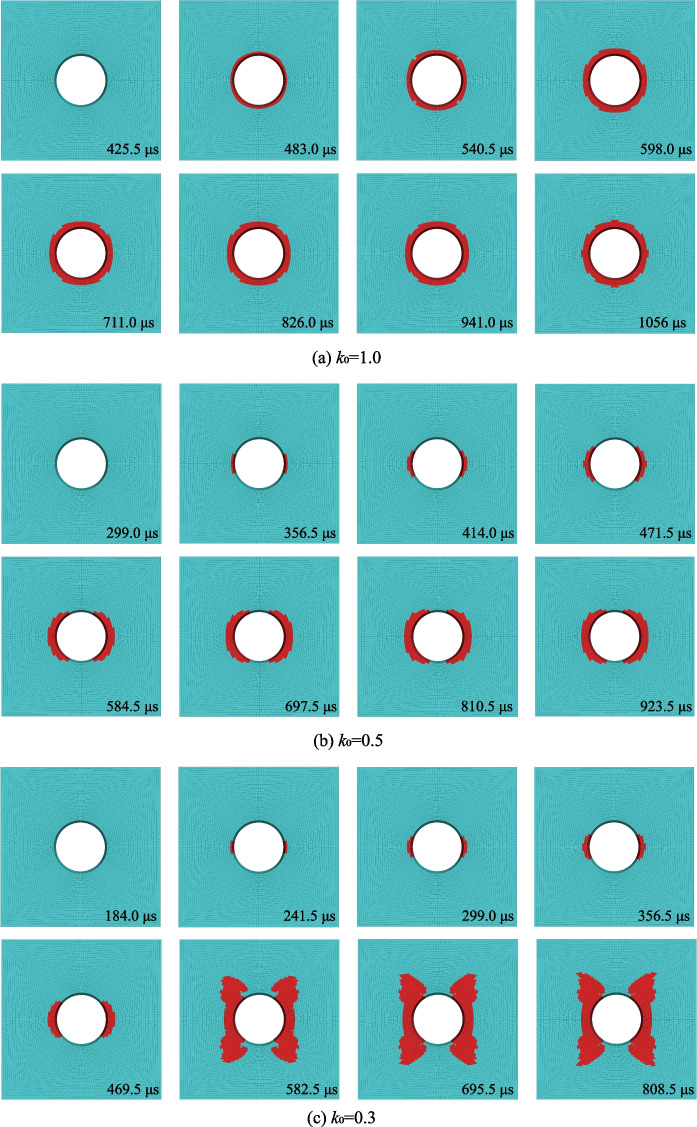
The evolution of EDZ during the transient tunnel excavation under different *k*_0_

Figure 19 shows the evolution of EDZ during transient excavation under three different lateral coefficients (i.e., *k*_0_ = 1.0, 0.5, and 0.3). The shear failure dominates the damage/plastic zone of the model, and the number of the shear failure zones (e.g., *sf*) increases with the decrease of *k*_0_. When *k*_0_ = 1.0, the geometry of the shear failure zone is of a ring shape. It changes from the ring shape to the egg shape when *k*_0_ drops to 0.5, and the shear failure at both the top and the bottom of the excavated tunnel disappears. For *k*_0_ = 0.3, the growth of EDZ at the initial stage is similar to when *k*_0_ = 0.5. However, when *k*_0_ = 0.3, its geometry finally turns to an X-shape, and the cumulative *sf* is much bigger than when *k*_0_ = 0.5.

**Fig. 20:**
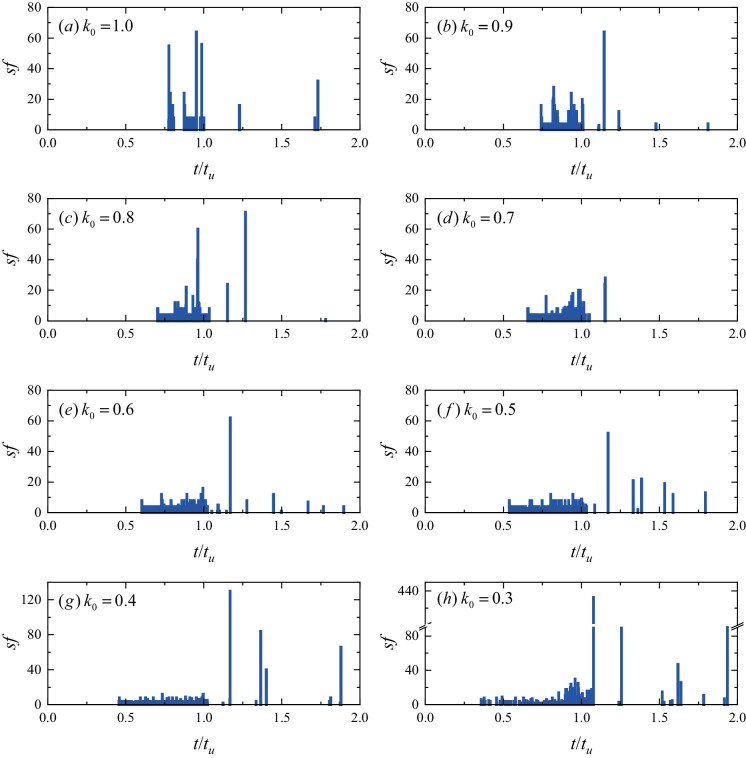
The accumulative number of shear failure zones *sf* after the transient unloading under different *k*_0_

To evaluate the influence of the lateral pressure coefficients on the failure process of the transient excavated tunnel, the accumulative number of the shear failure zones (*sf*) after the transient unloading under different lateral pressure coefficients *k*_0_ is shown in Fig. 20. When *k*_0_ is larger than 0.7, the failure of the surrounding zones is primarily concentrated in the period of 0.5 < *t*/*t*_u_ < 1.0 in Stage I (*t*_u_ is the total duration of Stage I) and the cumulative *sf* is almost constant in the subsequent duration. When *k*_0_ decreases from 0.6 and 0.3, the failure events start to be observed in Stage II (1.0 < *t*/*t*_u_ < 2.0) and are found to be more serve than in Stage I, e.g., the *sf* in Stage I is around 10, and the maximum *sf* reaches 80 in Stage II when *k*_0_ = 0.4. The failure initiation moment *t*_0_ decreases as the *k*_0_ decreases, indicating that the lateral pressure coefficient significantly influences the failure behavior of the surrounding rocks. Our numerical result may provide new insights for predicting the occurrence of rockburst (i.e., the site and magnitude of rockburst) and the type of rockburst i.e., the immediate rockburst (Feng et al 2015, 2016) and the time-delayed rockburst (Ma et al 2015; Liu et al 2016) in deep rock engineering. We are subsequently working-out in finding supporting evidence in other relevant studies to support this conclusion and account for the mechanisms of rockburst with the in-situ stress states of tunnels (i.e., the stress value and the lateral pressure coefficient). And hence, the design of the support of the excavated tunnel should be varied with the lateral pressure lateral coefficient *k*_0_ due to the discrepancy of the predicted EDZ (Fig. 19). For example, for a tunnel subjected to the hydrostatic stress (*k*_0_ = 1.0), the support should cover the whole tunnel face. The potential failure areas should be gradually transferred to the vaults of the tunnel as the *k*_0_ decreases, and special attention should be paid to the top and bottom of the tunnel to prevent the formation of tensile failures. Another important finding is that the severity of the transient unloading of the tunnel increases with the decrease of the lateral pressure coefficient (e.g., from 1.0 to 0.3), and the dramatic destruction of the surrounding rocks is prone to occur after the transient unloading is completed under a low *k*_0_ (< 0.7). Moreover, the dynamic responses (stress redistributions, particle displacements and vibrations) of deep transient excavated tunnels under various lateral pressure coefficients *k*_0_ values also indicate that large deformation and vibration are more prone to occur at squeezing tunnels that have low *k*_0_ values. Therefore, an efficient support system is needed to restrain the expansion of the EDZ and to reduce the risk of tunnel destruction hazards.

## Conclusions

This study adopted a new experimental method for simulating the transient excavation for deep-buried tunnels to investigate the dynamic mechanism of a transient excavated tunnel under different lateral pressure coefficients (*k*_0_ = 1.0, 0.8, 0.6, 0.4, 0.2). The experimental results show that the radial stress of the surrounding rocks decreases after transient excavation while the tangential stress change depends on the value of *k*_0_ and the position of the measuring point (i.e., the top/bottom or the vaults of the tunnel). The variation of tangential displacement of the surrounding rocks is much smaller than the radial displacement. The radial displacement on the top of the tunnel is negative and decreases with the distance between the measuring points to the tunnel center. Additionally, the horizontal radial displacement of P5 is positive (rightwards) and becomes negative when *k*_0_ is smaller than 0.6. The impact wave and the transient unloading wave can be detected in the vibration velocity spectrum, and the two components can be separated in the amplitude-frequency spectrum through FFT. The PPV of the vertical measuring points (i.e., P0–P4) was found to decrease with the increase in the propagation distance, while the PPV of P5 turns negative when *k*_0_ is smaller than 0.6. The amplitude of the amplitude-frequency spectrum decreases as the distance between the measuring point and the tunnel center increase. Furthermore, the low-frequency components in the frequency-amplitude spectrum increase significantly as the lateral pressure coefficient decreases.

The dynamic Mohr–Coulomb criterion is developed by considering the rate dependency correction on the strength parameters (i.e., the tensile strength ${\sigma }_{t}$ and cohesion *c*). Moreover, it was then adopted to study the dynamic failure mechanism of a transient excavated tunnel model in FLAC3D through Fish programming. The numerical results suggest that the EDZ of a transient excavated tunnel is dominated by shear failure, and the number of the shear failure zones *sf* would increase with the decrease of *k*_0_ and the geometry of the EDZ changes from ring-shape to egg-shape and X-type shear with the decrease of *k*_0_ accordingly. The evolution of the EDZ of the surrounding rocks is mainly distributed in the stress redistribution stage (Stage I) under a high *k*_0_ (from 1.0 to 0.7), while dramatic destruction of the surrounding rocks (e.g., rockburst) is more prone to occur after the finish of transient unloading under a low *k*_0_ (≤ 0.6). Therefore, timely support is needed to restrain the expansion of the EDZ and to reduce the risk of tunnel destruction hazards.

## Data Availability

The data that support the findings of this study are available from the corresponding author upon reasonable request.
